# Green Solvents for the Liquid Phase Exfoliation Production of Graphene: The Promising Case of Cyrene

**DOI:** 10.3389/fchem.2022.878799

**Published:** 2022-04-11

**Authors:** João Fernandes, Siva Sankar Nemala, Giovanni De Bellis, Andrea Capasso

**Affiliations:** ^1^ International Iberian Nanotechnology Laboratory, Braga, Portugal; ^2^ Department of Astronautical, Electrical and Energy Engineering, Sapienza University of Rome, Rome, Italy; ^3^ Research Center on Nanotechnology Applied to Engineering of Sapienza (CNIS), Sapienza University of Rome, Rome, Italy

**Keywords:** 2D materials, solution processing method, sonication, high-shear mixing, inkjet printing, environmental risks, sustainability

## Abstract

The liquid phase exfoliation (LPE) of graphite has allowed to produce graphene materials on a large scale and at a reasonable cost. By this method, stable dispersions, inks and liquid suspensions containing atomic-thick graphene flakes with tailored concentrations can be produced, opening up applications in a wide range of cutting-edge technologies such as functional coatings, printed and flexible electronics, and composites. However, currently established LPE techniques raise several health and environmental risks, since unsafe and toxic solvents (such as NMP, DMF, and DMSO) are often regarded as the most effective liquid media for the process. Therefore, it appears necessary to unlock eco-friendly and sustainable methods for the production of graphene at an industrial scale. This review focuses on the latest developments in terms of green solvents for LPE production of graphene. We highlight the use of a new green solvent, Cyrene, and its performance when compared to conventional solvents.

## Introduction

As the archetypal two-dimensional material, graphene has been the proposed material in the last decade for several technologies such as wearable/flexible electronics (Tan et al., 2017), structural and multifunctional nanocomposites (Wang et al., 2021), energy storage (Li and Zhi, 2018), strain sensors (Mehmood et al., 2020), water treatment (Bhol et al., 2021) and biomedical devices (Yang et al., 2013). A scalable mass production of highly pure graphene at low cost is the prerequisite for the commercialization phase. Among the many production methods, liquid phase exfoliation (LPE) allows to obtain liquid dispersions of graphene flakes with high yield. LPE technique was initially reported in 1989 for MoS_2_ and WSe_2_ (Gutiérrez and Henglein, 1989) and translated to graphene in 2008, demonstrating an affordable production of 2D materials in large quantities (Hernandez et al., 2008). LPE graphene flake dispersions are suitable for several applications, such as flexible, transparent, and printable electronics (Secor et al., 2013; Secor et al., 2015; Li et al., 2018; Shin et al., 2018). Usually, LPE identifies a group of approaches where natural and synthetic bulk materials are directly exfoliated into their corresponding isolated layers in a liquid medium, using the energy provided by different techniques: ultrasonication (Turner et al., 2019), wet ball-milling (Zhao et al., 2010), electrochemical, micro-fluidization (Xu et al., 2018), and high-shear mixing force (Paton et al., 2014), wet-jet milling (Del Rio Castillo et al., 2018) and high-pressure system (using an airless paint sprayer) (Nemala et al., 2018). These approaches can be executed in a variety of liquid solvents, including water (frequently mixed with surfactants), organic solvents, ionic liquids, oils, and salts (Xu et al., 2018). The general LPE process consists of three steps: intercalation, exfoliation and separation (Li et al., 2020). The solvent is a crucial factor in the exfoliation process, and to be effective it should fulfil three main requirements: 1) transmit the exfoliating power efficiently, 2) minimize the energy needed to disrupt the van der Waals forces among layers and 3) stabilize the exfoliated layers by providing steric hindrance to prevent re-agglomeration (Banavath et al., 2021). We will start this mini review by giving an overview of the most effective solvents for LPE of graphite. Although commonly used, these solvents entail severe health and environmental risks and should be replaced to reach a sustainable commercialization phase. The search for “green” solvents thus appears pivotal. By analyzing recent literature, we will describe the most representative green options to make stable graphene-based dispersions at high yield. As a case study, we will focus on dihydrolevoglucosenone (trademarked as Cyrene), which can be currently regarded as the most promising green solvent for LPE graphene.

## Towards Green Solvents for the LPE of Graphite

An ideal solvent for the exfoliation of graphite into graphene should meet several key requirements. In general, an ideal solvent would allow the complete exfoliation of graphite, leaving no un-exfoliated flakes in the sediment. The Hansen solubility parameters offer a framework to predict if and how a material will disperse in a particular solvent and form a solution (Charles, 2007). The surface tension of the solvent and graphite should ideally match to stabilize the graphene flakes in the dispersion after the exfoliation, preventing their re-agglomeration (Shen et al., 2015). For these reasons, researchers have originally selected solvents that matched as much as possible the Hansen solubility parameters and surface tension value of graphite (Hernandez et al., 2008; Capasso et al., 2015; Shen et al., 2016; Xu et al., 2018). The dynamic viscosity of the solvent is another important parameter in terms of exfoliation efficiency and stability. In principle, a high viscosity would be beneficial for the LPE process, increasing the exfoliation yield and decreasing the defect density and sedimentation rate (Manna et al., 2016; Salavagione et al., 2017; Simfukwe et al., 2017). However, a threshold must be set for practical applications, since an excessive viscosity favors the stable suspension of large agglomerates/particles during the centrifugation step, thus preventing the separation from thinner and lighter flakes (Backes, 2020). As a last consideration, a LPE solvent should feature a low boiling point to allow an easy removal of any solvent residue, which might degrade the properties of graphene (especially in terms of electrical conductivity) (Neill, 2009).

Conventional solvents for the LPE of graphite (surface tension ∼55 mN m^−1^ (Bonaccorso et al., 2012)) exhibit a surface tension ranging within 40–50 mN m^−1^ and Hansen solubility parameters close to those of graphite (*δ*_D_ = 18.0 MPa^0.5^, *δ*_P_ = 9.3 MPa^0.5^, *δ*_H_ = 7.7 MPa^0.5^) (Hernandez et al., 2010). Within this range, several highly polar solvents were selected, including N-methylpyrrolidone (NMP), N,N-dimethylformamide (DMF), dimethylsulfoxide (DMSO), N,N-dimethylacetamide (DMAC), and γ-butyrolactone (GBL) (Güler et al., 2021). Non-polar solvents such as ortho-dichlorobenzene (DCB) were also reported to produce homogeneous graphene dispersions (Güler et al., 2021). In general, amine-based solvents such as NMP and DMF are the most effective in producing crystalline, oxygen-free graphene flakes (Güler and Sönmez, 2020). Hernandez et al. originally reported the production of stable dispersions of few-layer graphene in NMP (Hernandez et al., 2008; Xu et al., 2018). The initially reported concentration of 0.01  mg mL^−1^ has been gradually increased above 1 mg ml^−1^ by several groups with longer sonication times (Khan et al., 2010; Wang et al., 2012; Wu et al., 2014). Successful exfoliation and stable dispersions were also reported in DMF and DMSO, with concentrations similar to those obtained in NMP (Coleman, 2013; Xu et al., 2018; Trusova et al., 2021; Vacacela Gomez et al., 2021). Although the exfoliation is effective, NMP, DMF, and DMSO have high boiling points which cause issues in the removal of solvent residues. More importantly, these solvents present severe health risks. In 2008, NMP and DMF were classified as Substances of Very High Concern. According to the European REACH (Registration, Evaluation, Authorisation and Restriction of Chemicals) regulation (Regulation No 1907/2, 2022), several restrictions were applied regarding their use or import to Europe. Same warnings were raised in the USA. DMSO has also recently raised serious safety concerns, after several studies have demonstrated both the toxicity on retinal neuronal cells (Galvao et al., 2014) and the “extreme changes in micro RNAs and alterations in the epigenetic landscape”, in both cardiac and hepatic micro-tissues, even for concentrations as low as 0.1% (Verheijen et al., 2019).

In this context, current solvents for LPE graphene appear as a limiting factor in the long-term development and sustainability of the production. Safety concerns also demand the need for impractical and expensive equipment (*e.g.*, safety equipment, fume-hoods, exhausts, etc.), with a direct impact on the production cost. In order to scale-up the process and approach an industrial production, the identification of environmentally safe solvents that do not raise health risks is thus imperative. These solvents should be efficient for the exfoliation process, while having a moderate cost. A low boiling point is also a desired feature. Such characteristics would at once minimize the ecological impact and lower the production complexity and cost of the production of graphene. Capello et al. proposed a framework for a comprehensive assessment of how “green” a solvent is (Capello et al., 2007). The authors used a complementary, multi-criteria evaluation: They combined EHS (environment, health and safety) considerations on the inherent hazards of a solvent, and a LCA (life-cycle-assessment) that quantifies the energy use connected to solvent production and disposal/treatment as waste (Capello et al., 2007). According to this definition, low-boiling-point solvents such as acetone and isopropyl alcohol (61°C and 56°C, respectively) can be considered green alternatives (Capello et al., 2007). They have been previously used to disperse graphene at low concentration (few μg mL^−1^) (Hernandez et al., 2010). However, these solvents have low flash points (12–13°C), which raise safety concerns for industrial use. Cyclohexanone and cyclopentanone have also been previously proposed as green and bio-based LPE solvents, but they present similar issues (flash point of 44°C and 31°C, respectively) (Hernandez et al., 2010).

Other green alternatives are represented by aqueous media with surfactants (*e.g.*, sodium dodecylbenzenesulfonate (Lotya et al., 2009), sodium cholate, (Green and Hersam, 2009), and sodium deoxycholate (Hasan et al., 2010)), and/or polymers (e.g., Pluronic^®^ (Seo et al., 2011)) useful to overcome surface tension mismatch (water has a surface tension of 72 mN m^−1^). Surfactant-assisted exfoliation in aqueous media is one of the most suitable alternatives to achieve high-quality graphene at high concentrations (Zhang et al., 2018). Green et al. prepared stable dispersions of graphene using sodium cholate (SC) as a surfactant in aqueous medium, yet achieving a low concentration (10 μg ml^−1^) (Green and Hersam, 2009). In an analogous study, dispersions in water and SC were prepared by tip-sonication up to 7 mg/ml concentration using longer exfoliation times (96 h) and high SC concentration (5 mg/ml) (Nawaz et al., 2016). Coleman et al. made graphene dispersions in a range of aqueous solutions (containing ionic or non-ionic surfactants) at similar concentrations (10–30 μg ml^−1^) (Smith et al., 2010). Dispersions of graphene flakes (average lateral size of ∼1 μm and layer number of ∼4.5) in water and Triton X-100 (a non-ionic surfactant) were also obtained by tip-sonication at a concentration of 0.54 mg/ml (Arao and Kubouchi, 2015). Nevertheless, residuals arising from the surfactants are known to reduce the quality and the electrical conductivity of the exfoliated flakes, thus limiting their use in electronic applications. To overcome this issue, mixtures of solvents were also considered (Zhou et al., 2011), such as water/isopropyl alcohol (Halim et al., 2013) and water/ethanol (Capasso et al., 2015). However, these mixtures also do not allow to obtain dispersions at high concentration.

**FIGURE 1:**
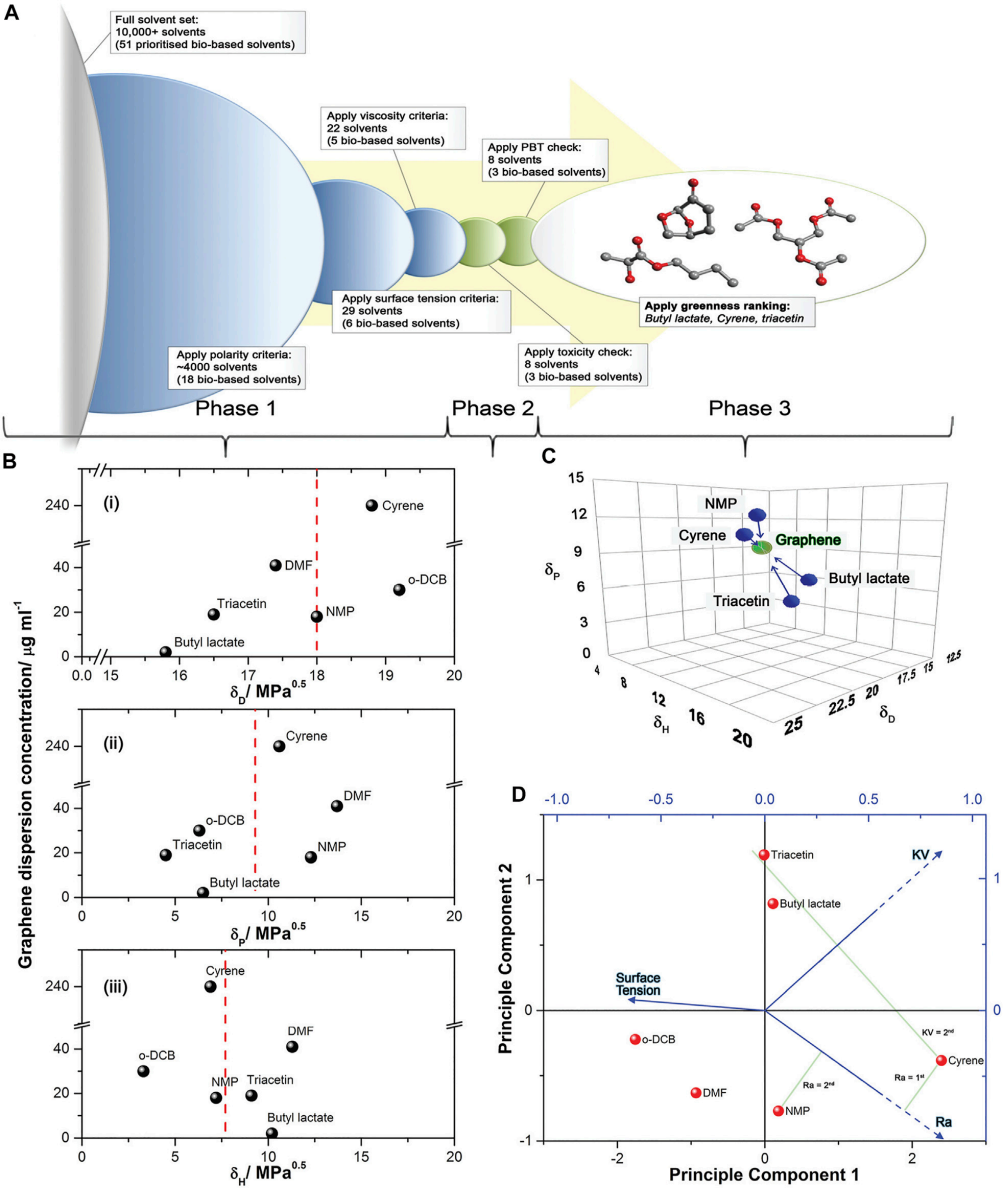
The application of solvent selection criteria for optimizing graphene dispersions. **(A)** Illustration of the solvent selection steps applied for the computational screening of suitable solvents. **(B)** Graphene dispersion concentration as a function of (i) dispersive, δD (ii) polar, δP and (iii), hydrogen-bonding, δH Hansen solubility parameters, with the dashed red line being indicative of ideal graphene properties. NMP, DMF and DCB are shown as reference. **(C)** Hansen solubility map showing the similarity of the final bio-based solvent candidates (and NMP) to graphene in terms of their polarity. The Hansen radius (Ra) is the radius of the sphere in the Hansen space, where each axis corresponds to one solubility parameter. **(D)** Principle Component Analysis (PCA) bi-plot for candidate solvents (including NMP, DCB and DMF for reference) with vectors indicating surface tension, kinematic viscosity (KV) are and Hansen radius (Ra). Reproduced with permission from Ref (Salavagione et al., 2017). Copyright ^©^ The Royal Society of Chemistry 2017.

Therefore, there is a need for new green solvents which fulfill the aforementioned requirements. Salavagione et al. identified efficient solvents by computational methods. The authors applied criteria including polarity, surface tension, viscosity, toxicity and “greenness” to evaluate and shortlist a solvent set of more than 10,000 (Figure 1A). Three bio-based solvents fulfilling the criteria were selected and tested experimentally: cyrene, triacetin, and butyl lactate (Salavagione et al., 2017). Figure 1B shows the Hansen solubility parameters δ_D_, δ_P_ and δ_H_ (measuring the energies from dispersion forces, dipolar intermolecular forces and hydrogen bonds between molecules, respectively). These three parameters can be set as coordinates for a point in a three dimensional graph known as the Hansen space. The Hansen solubility space in Figure 1C shows how bio-based candidates (and NMP) and graphene compare. Triacetin and butyl lactate have appropriate parameters, with the exception of δ_P_. Nonetheless, they have high boiling points and led to dispersions with low concentration.

Other green alternatives [triethanolamine (Chen et al., 2017)—TEA–and urea aqueous solutions (He et al., 2015)] have been tested for the LPE of graphite. In Table 1, we summarize the features of these green solvents, comparing them to conventional ones. In the single report currently available on the use of TEA, the authors obtained dispersions of crystalline graphene flakes with high stability (over 9 months) (Chen et al., 2017). However, TEA can induce detrimental chemical modifications in the flakes, possibly due to functionalization (Song et al., 2015; Ryu et al., 2017; Paolucci et al., 2020). Furthermore, its very high viscosity precludes the deposition of the dispersion by versatile techniques, such as inkjet printing (usually requiring 1–10 cP) (Paolucci et al., 2020). As a next alternative, urea aqueous solution has also been tested and led to crystalline flakes and 15-days stability (He et al., 2015; Hou et al., 2019). However, this solvent is theoretically not ideal for graphite exfoliation, since both the surface tension and Hansen solubility parameters are higher than desired (especially the δ_H_ and δ_P_ components). As a proof of that, a very low yield (2.4%) was reported (Paolucci et al., 2020). Methyl-5-(dimethylamino)-2-methyl-5-oxopentanoate (Rhodiasolv Polarclean) is a polar solvent that was also tested in sonication-assisted LPE of graphite, MoS_2_ and WS_2_. It showed good performance, with a ∼350% higher amount of few-layer nanosheets (<5 nm thickness) and 10 times lower defect density with respect to NMP (Paolucci et al., 2020). However, its high boiling point represents a pivotal drawback. As for TEA, we note that there is just a single report available on the use of Polarclean, making it difficult to draw a conclusive comparison to the other solvents. Overall, these results point out that TEA, urea and Polarclean are not suited to a scalable production of graphene.

**TABLE 1:** Summary of the features of conventional (NMP, DMF and DMSO) and green solvents used for LPE of graphite.

Solvent	Surface tension (σ_S_ [mN m^−1^])∼55^a^	Dynamic viscosity@ 25°C (cP)	Boiling Point (°C)	Hansen solubility parameters			Cost^b^ (for 1 L) €	Dispersion Concentration (mg ml^−1^)
				δ_d_ [MPa^1/2^] 18.0^a^	δ_P_ [MPa^1/2^] 9.3^a^	δ_H_ [MPa^1/2^] 7.7^a^		
NMP	**40.1** (Paolucci et al., 2020)	**1.65** (Pourabadeh et al., 2020)	**202** (Sherwood et al., 2014)	**18.0** (Hernandez et al., 2010)	**12.3** (Hernandez et al., 2010)	**7.2** (Hernandez et al., 2010)	**147.00**	**1.50** (Wu et al., 2014)
DMF	**37.1** (Paolucci et al., 2020)	**0.9** (Alam et al., 2019)	**153** (Sherwood et al., 2014)	**17.4** (Hernandez et al., 2010)	**13.7** (Hernandez et al., 2010)	**11.3** (Hernandez et al., 2010)	**161.00**	**1.30** (Trusova et al., 2021)
DMSO	**42.9** (Yaws, 2014)	**1.99** (Zhang et al., 2017)	**189** (Sherwood et al., 2014)	**18.4** (Charles, 2007)	**16.4** (Charles, 2007)	**10.2** (Charles, 2007)	**88.10**	**0.03–0.2** (Chouhan et al., 2020)
Triacetin	**32.6** (Yaws, 2014)	**16.31** (Rodríguez et al., 1994)	**258**	**16.5** (Salavagione et al., 2017)	**4.4** (Salavagione et al., 2017)	**9.0** (Salavagione et al., 2017)	**57.40**	**0.02** (Salavagione et al., 2017)
Butyl lactate	**29.2**	**3.9**	**188**	**15.7** (Salavagione et al., 2017)	**6.4** (Salavagione et al., 2017)	**10.2** (Salavagione et al., 2017)	**190.80**	**0.002** (Salavagione et al., 2017)
TEA	**45.9** (Paolucci et al., 2020)	**607** (Maham et al., 2002)	**89.28**	**17.3** (Paolucci et al., 2020)	**7.6** (Paolucci et al., 2020)	**21.0** (Paolucci et al., 2020)	**95.70**	NA
Urea aqueous solutions (30%)	**74.0** (Paolucci et al., 2020)	**1.36** (Kawahara and Tanford, 1966)	NA	**17.0** (Paolucci et al., 2020)	**16.7** (Paolucci et al., 2020)	**38.0** (Paolucci et al., 2020)	NA	**0.15** (He et al., 2015)
Polarclean	**38.0** (Paolucci et al., 2020)	**9.78**	**273.7**	**15.8** (Paolucci et al., 2020)	**10.7** (Paolucci et al., 2020)	**9.2** (Paolucci et al., 2020)	NA	**0.3** (Paolucci et al., 2020)
Cyrene	**72.5** (Salavagione et al., 2017)	**14.5** (Paolucci et al., 2020)	**227** (Sherwood et al., 2014)	**18.7** (Salavagione et al., 2017)	**10.8** (Salavagione et al., 2017)	**6.9** (Salavagione et al., 2017)	196.00	**0.7** (Salavagione et al., 2017)

a Reference values of graphite.

b From Sigma Aldrich.

As shown in Figure 1, Cyrene has the smallest Hansen radius (2.2 MPa^0.5^), demonstrating the greatest affinity to graphite (as shown in Figures 1C,D). It has the second highest kinematic viscosity (Figure 1D), high enough to guarantee a high exfoliation yield, while preventing severe sedimentation over time. This value is also suitable to allow the deposition by techniques such as inkjet printing. Cyrene has a higher surface tension than other conventional solvents, which should be evaluated in terms of dispersion stabilization. Recent reports indicate Cyrene as a promising green solvent for LPE of graphite, so we analyze it in detail in the following section.

## Case Study: Cyrene as the Most Advanced Green Solvent for LPE of Graphite

Cyrene (dihydrolevoglucosenone, C_6_H_8_O_3_) is a bio-based solvent derived (Pecka et al., 1974) in two steps from cellulose via levoglucosenone (biomass) (De bruyn, 2016), a process that guarantees at once low environmental impact and economic feasibility. In 2014, dihydrolevoglucosenone was marketed by Australian biotechnology company Circa Group in conjunction with Professor James Clark (University of York’s - Green Chemistry Centre of Excellence) as Cyrene. Nowadays, Cyrene is commercialized by Merck. Composed of two fused rings, Cyrene does not present the amide functionality (typical of NMP and DMF) that is linked to reproductive toxicity effects (Salavagione et al., 2017). It neither contains any chlorine groups, which are usually responsible for end-of-life pollution issues. When incinerated, Cyrene yields only carbon dioxide and water as byproducts: This is a major difference over NMP, which liberates NO_x_ when decomposed. Also, Cyrene has very low acute and aquatic toxicity with LD_50_ (lethal dose, 50%) and EC50 (effective concentration, 50%) values of >2000 mg kg^−1^ and >100 mg L^−1^, respectively. Overall, Cyrene is biodegradable and not mutagenic. Although it has a rather high boiling point (227°C), its low flash point (108°C, lower than several oxygenated solvents, such as alcohols and ketones) makes it safe to handle.

Salavagione et al. first demonstrate the preparation of LPE graphene in Cyrene. After 2 h of bath sonication, the dispersion showed a final concentration ∼0.7 mg ml^−1^, with a very high yield (∼48%). These values are larger than those obtained by most conventional organic solvents, also requiring more complex LPE procedures (Lavin-Lopez et al., 2016). In their analysis, 92.5% of the dispersed flakes were few-layer (more than 10), 75% within five layers, and 7.5% monolayer (final average of 4.5 layers). In similar bath sonication experiments, Gharib et al. obtained a 6 times higher concentration with respect to NMP and DMF (Gharib et al., 2017). Tkachev et al. proposed the preparation of a graphene-based inks in Cyrene by a combination of two LPE methods (*i.e.*, tip-sonication and high-shear mixing). The authors produced highly concentrated dispersions (up to 3.70 g L^−1^) of few-layer graphene flakes (three to five layers) with mean lateral size of ∼200 nm (Tkachev, 2021). Pan et al. developed an environmentally friendly, sustainable, low-cost graphene-based ink in Cyrene with concentration up to 10 mg ml^−1^, by using sonication assisted exfoliation. The authors added cellulose acetate butyrate (CAB) as a stabilizing agent to achieve even a higher concentrated ink (70 mg ml^−1^) (Pan et al., 2018) of multilayer graphene flakes (thickness ∼5 nm) with lateral size of a few µm.

In terms of applications, Pan et al. screen-printed electrodes from graphene inks in Cyrene and NMP. They obtained analogous sheet resistance values (∼1 Ω □^−1^) using inks produced with significantly different sonication times (8 h for Cyrene *vs*. 48 h for NMP). The electrical conductivity of dried and compressed graphene laminates from Cyrene ink (8 h sonication) was 7.13 × 10^4^ S m^−1^. These results pave the way to low-cost, screen-printable graphene-based wearables for Internet of Things applications, such as healthcare and wellbeing monitoring (Pan et al., 2018). Tkachev et al. prepared graphene-based inks in Cyrene to spray-coat flexible semi-transparent electrodes with high optical transmittance (78%) and low sheet resistance (290 Ω □^−1^). They embedded such electrodes in a working prototype of a multi-touch screen with a high signal-to-noise ratio (14 dB). These results illustrate a potential pathway toward the integration of LPE-graphene in commercial flexible electronics (Tkachev, 2021). Hassan et al. proposed a green ink combining Cyrene and ethyl cellulose (polymeric binder that helps lowering sheet resistance by enhancing connectivity and filling the gaps). They used it to fabricate (by 3D extrusion printing) low-cost patterned electrodes for volatile organic compounds detection fabricated. The devices showed a resistivity as low as 70 Ω cm and high sensitivity to organic compounds (*i.e.*, acetone, ethanol, and methanol). In particular, the device showed a high sensitivity towards ethanol (Hassan et al., 2021). These case study results suggest that Cyrene based graphene inks are more stable and suitable than the currently employed solvents for commercial applications, without any toxicity issues.

## Conclusion

In summary, there is an urgent need of replacing conventional solvents like NMP and DMF for the liquid phase production of graphene, in order to reduce health and environmental issues and enable a sustainable industrial production. We have presented the most viable “green” solvents in the field, comparing their different properties and their effectiveness (in terms of concentration and yield) as exfoliation media. Among the possible options, Cyrene appears as the most promising green solvent for LPE techniques. The performance of Cyrene for the exfoliation of graphite was analyzed, also focusing on research literature reporting graphene-based devices prepared using this solvent. This mini-review sheds light on a sustainable solution processing methods for graphene, but the findings could be translated to other layered 2D materials, such as hBN, transition metal dichalcogenides and MXenes.
